# Eye movements reflect active statistical learning

**DOI:** 10.1167/jov.24.5.17

**Published:** 2024-05-31

**Authors:** József Arató, Constantin A. Rothkopf, József Fiser

**Affiliations:** 1 Department of Cognitive Science, Central European University, Vienna, Austria; 2 Center for Cognitive Computation, Central European University, Vienna, Austria; 3 Vienna Cognitive Science Hub, University of Vienna, Vienna, Austria; 4 Center for Cognitive Science & Institute of Psychology, Technical University of Darmstadt, Darmstadt, Germany; 5 Frankfurt Institute for Advanced Studies, Goethe University, Frankfurt, Germany

**Keywords:** active learning, eye movements, statistical learning, implicit learning, information search

## Abstract

What is the link between eye movements and sensory learning? Although some theories have argued for an automatic interaction between what we know and where we look that continuously modulates human information gathering behavior during both implicit and explicit learning, there exists limited experimental evidence supporting such an ongoing interplay. To address this issue, we used a visual statistical learning paradigm combined with a gaze-contingent stimulus presentation and manipulated the explicitness of the task to explore how learning and eye movements interact. During both implicit exploration and explicit visual learning of unknown composite visual scenes, spatial eye movement patterns systematically and gradually changed in accordance with the underlying statistical structure of the scenes. Moreover, the degree of change was directly correlated with the amount and type of knowledge the observers acquired. This suggests that eye movements are potential indicators of active learning, a process where long-term knowledge, current visual stimuli and an inherent tendency to reduce uncertainty about the visual environment jointly determine where we look.

## Introduction

Across their awake lives, people make two or three saccades per second, which fundamentally determines the sensory information reaching their high-level cognition during fixations and in turn, what they will remember about a visual scene (Findlay & Gilchrist, 2003). Conversely, observers’ knowledge about a visual scene and its context influences where they look thus creating a bi-directional link between eye movements and memory (Chun & Turk-Browne, 2007; Irwin & Gordon, 1998, Schütz, Braun, & Gegenfurtner, 2011). Theoretically, such a bidirectional interaction represents a special case of “active learning,” a computational framework in which the active learner can achieve better learning with fewer training samples because of selectively choosing the subset of data from which it predicts to learn best (Settles, 2009). In the case of vision, this refers to the process in which the sensors (i.e., the eyes) are directed to particular locations selectively to choose what new information to explore based on the already accumulated (implicit or explicit) knowledge from previous experience thereby achieving more effective learning (Gottlieb, 2012). As the obtained new information at any fixation is immediately available and incorporated into the existing knowledge base, the next fixation is already influenced by what has been learned in the previous fixation.

Although this bidirectional interaction between sampling visual information through eye movements and learning is theoretically well established, there is surprisingly little empirical evidence supporting such an ongoing process. Eye movements as information queries have mostly been studied in scenarios where the information about the environment had to be collected on a short timescale (<1–2 seconds– within a single trial), in a paradigm called “active sensing” (Yang, Lengyel, & Wolpert, 2016; Yang, Wolpert, & Lengyel, 2016). A number of studies have demonstrated active sensing in human behavior by showing that the selection of fixation targets to reduce uncertainty is near optimal (Hoppe & Rothkopf, 2019; Najemnik & Geisler, 2005; Yang, Lengyel, et al., 2016), although remarkable failures have also been reported (Morvan & Maloney, 2012). However, whether human eye movements perform “active learning”—continuously incorporating information about structural aspects of the environment manifested not just within a trial but on longer timescales into the immediate control of eye movements—is largely unknown. Assessing sensitivity even to simple statistical structures, when visual scene regularities are only detectable across trials, such as higher target probability at certain areas of the screen, resulted in mixed findings in visual search studies not measuring eye movements (Chun & Jiang, 1998; Geng & Behrmann, 2005; Kunar, Flusberg, Horowitz, & Wolfe, 2007). Similarly mixed results were obtained in studies using eye tracking: some suggested that people were capable of incorporating simple statistical regularities of their visual environment during saccadic target selections (Boettcher, Shalev, Wolfe, & Nobre, 2022; Jiang, Won, & Swallow, 2014; Jones & Kaschak, 2012; Talcott & Gaspelin, 2020), whereas others reported failure of doing so (Morvan & Maloney, 2012; Paeye, Schütz, & Gegenfurtner, 2016; Walthew & Gilchrist, 2006; Droll, Gigone, & Hayhoe, 2007), presumably because of large individual differences (Irons & Leber, 2016).

In case of more complex statistical structures, a substantial body of work has investigated how perceptual experience at different time scales affects eye movement control (Findlay & Gilchrist, 2003; Hayhoe & Ballard, 2005; Kowler, 2011; Yarbus, 1967). For example, it has been reported that gaze biases can emerge from a lifetime of experience: anticipating a ball's trajectory in sports (Brockmole & Henderson, 2006; Land & McLeod, 2000), the tendency to perform visual search from left to right (Spalek & Hammad, 2005), or using acquired semantic knowledge (Võ & Wolfe, 2013) or meaning in real-world scenes (Castelhano & Heaven, 2011; Henderson, Hayes, Rehrig, & Ferreira, 2018). It has also been shown that information from the last couple of minutes, such as object co-occurrences (Brockmole & Henderson, 2006; Mack & Eckstein, 2011) and episodic memory (Li, Pilar Aivar, Tong, & Hayhoe, 2018) can guide visual search. However, all the above studies considered the effect of a particular, well defined explicit task (e.g. categorization, detection, visual search) with specific instructions, and they focused on end results instead of the process of learning: they showed that after practice, eye movements became more related to diagnostic features (e.g.: identity, location) of the task. These results do not address the questions whether this is a bi-directional interaction that occurs continuously and gradually (Wolfe & Horowitz, 2017) and whether it can occur without any explicit task (Theeuwes, Bogaerts, & van Moorselaar, 2022) as it is assumed by active learning.

One issue hindering the progress in relating theoretical accounts of active learning to empirical investigations of the interaction between sampling visual information through eye movements and learning is the fact that the empirical studies predominantly used the theoretical frameworks of reinforcement learning (Dayan & Daw, 2008; Sutton & Barto, 2018) or supervised learning (Bishop & Nasrabadi, 2006; Schultz & Dickinson, 2000), in which a less or more specific feedback of the correct action or response is provided during learning. In contrast, active learning in sensory processing is fundamentally conceptualized as an unsupervised process, in which there is no explicit reward or feedback, beyond the possibility that learnable information in itself is rewarding for an active learner (Chun & Turk-Browne, 2007; Gottlieb, 2012; Gottlieb & Oudeyer, 2018; Nobre & Stokes, 2019; Settles, 2009). A study exploring the role of supervision in the use of spatial cues to facilitate target search found minimal effects on eye movements without feedback (Droll, Abbey, & Eckstein, 2009).

A suitable paradigm to study unsupervised active learning in the perceptual domain without explicit reward is the spatial version of visual statistical learning (Fiser & Aslin, 2001; Fiser & Aslin, 2005). In this version of statistical learning, observers are exposed to a stream of sensory stimuli (visual scenes composed of multiple simple shapes) without specific task instructions or feedback, and they automatically and implicitly pickup regularities defining the underlying structure of the scenes, whereas learning is measured after exposure with a familiarity test. In the familiarity test, true pairs are tested against foil pairs, which are assembled from the same shapes. Because in this paradigm, the only learnable regularity in the presented stream of scenes (beyond the identities of the randomized shape) is a spatial co-occurrence structure between the shapes imposed by the experimenter and unknown to the observer, learning across scenes can be precisely quantified on a subject-by-subject basis. However, this paradigm so far did not allow for measuring the interaction between sampling visual information through eye movements and learning.

To achieve this, we used a gaze contingent stimulus presentation for quantifiable measurement of the link between learning and information gathering by eye movements. We also manipulated the length of learning and the amount of explicit information about the underlying structure of the scenes provided at the beginning of the experiment to assess their influence on learning (Castro-Rodrigues et al., 2022). With this modified paradigm, we found that although detectable statistical learning could already occur without any noticeable effect on eye movements, a tight link emerged inevitably and gradually between information search as quantified by eye movement patterns and learning the structure. Importantly, this link emerged under both conditions: when explicit information about the structure of the scenes was provided explicitly, and also without any explicit information, when extended exposure was provided to the observers to gather sufficient information.

## General methods

### Participants

One hundred twenty participants naïve about the purpose of the study and about statistical learning were recruited via a local student organization and received monetary compensation for their participation. Forty participants were assigned to each experiment (Experiment 1: age: 25.5 ± 4.6 years, 13 male; Experiment 2: age: 22.1 ± 2.8 years, 13 male; Experiment 3: age: 23 ± 5.5 years, 10 male). One additional participant completed Experiment 2 but was excluded from the final sample, because upon completing the study revealed not being naïve about visual statistical learning. Otherwise, we checked for but did not remove outliers in our datasets (Supplementary Material S1 for control analysis). We chose a sample size larger than most previous statistical learning studies (Batterink, Reber, Neville, & Paller, 2015; Fiser & Aslin, 2001; Turk-Browne, Jungé, & Scholl, 2005) because we wanted to assess the variability in the individual learning performances. This sample size has more than 90% power with α = 0.05, assuming a 0.5 Pearson correlation between eye movements and learning.

### Procedure

The experiment was conducted in a dimly-lit and sound-attenuated room. A Tobii EyeX 60Hz eye tracker was calibrated using a seven-point calibration from a viewing distance of 60 cm. After calibration, participants completed ten six-second-long practice trials where randomly selected images of dogs were revealed in a gaze-contingent manner within the 3 × 3 grid (28.4° × 28.4° visual): the content of each cell was visible only when the observer's gaze fell within the central 5.7° × 5.7° of the cell (referred to as the “central region” of the cell) in two subsequent eye position samples (approximately 15 ms apart), otherwise the given cell was shown empty. The size of the central region was chosen to ensure an easy and well identifiable fixation on individual shapes. The trials in the learning phase of each experiment were also six seconds long, and they followed the same gaze-contingent rule as during practice.

After calibration and practice, but before the start of the main experiment, in Experiment 1 participants were instructed to explore the scenes and find pairs of shapes that always appear next to each other in a horizontal, vertical, or diagonal arrangement. They were also told that they would be questioned about the identity of the pairs afterward (“explicit instructions”). Participants had six seconds to explore each of the 144 scenes, presented in a random order, resulting in a total training time of approximately 16 minutes. All aspects of Experiments 2 and 3 were identical to those in Experiment 1 except for the lack of explicit instructions. In Experiments 2 and 3, before the start of the main experiment, participants were told to explore the scenes and pay attention to what they saw. They were also told that they would be tested on what they had seen after the exploration phase, but they were not informed either about any potential regularity or structure in the stimuli or about the nature of the subsequent test. This setup matched the canonical conditions of implicit visual statistical learning used in previous studies (Fiser & Aslin, 2001; Turk-Browne et al., 2005). Experiment 2 was the same length as Experiment 1, but in Experiment 3, the learning phase was double in length: each one of the 144 unique scenes was presented twice, once in each half of the experiment in a different random order. In Experiment 3, completing the learning phase took approximately 32 minutes, with a short break in the middle, where participants were kindly asked to continue paying attention.

Each trial started by a fixation cross appearing in one of the empty grid cells, where the observer had to fixate to initiate the trial. The position of the fixation cross was uniformly distributed across trials, appearing at the center of each cell of the 3 × 3 grid an equal number of times during the experiment in a random order. Unlike previous spatial statistical learning studies, the full scenes in these trials were never visible at once. Instead, individual shapes were revealed in a gaze-contingent manner, when the participants’ gaze was inside the central region of a cell. When participants looked at a cell containing the shape (two subsequent gaze samples fell inside the central region of a cell), the shape appeared at full contrast (within 50 ms—shorter than delays used in Somai, Schut, & Van der Stigchel [2020] or Hoppe & Rothkopf [2019]) as long as the participant's gaze was in the given cell, but gradually faded away becoming invisible within 1.5 seconds when the participant looked away to a different cell. This way, maximally two shapes of the scene were displayed at any given time and only one of them at full contrast. If the observer's gaze was in the mid-region of a cell not containing a shape in a given trial, a gray rectangle was revealed indicating that the cell was empty to reduce the observer's uncertainty whether s/he managed to fixate on the cell. These gray rectangles remained visible until the end of a trial, at which point they disappeared, thereby ensuring that the end of each trial was easily noticeable. Participants were free to visit or revisit with their gaze any of the cells during the trial. Six seconds after the end of a trial all shapes and gray rectangles disappeared, only the grid remained visible, and after a 500 ms inter-trial-interval, the next fixation-cross appeared at one of the cells to initiate the start of the next trial. This arrangement was adequate to handle the multiple requirements of measuring the participants‘ sampling strategies (Supplementary Material S9).

At the end of the learning phase, after a short break, a two-interval-forced-choice test session followed, with trials in which participants were instructed to select the more familiar of the two pair combinations presented based on what they had seen during the learning phase. For the test, six foil pairs (with two shapes that never appear in the presented arrangement during learning) were created from the original shapes and those were tested in a fully counterbalanced manner against each of the real pairs of the inventory, resulting in 36 test trials presented in a random order (as in Experiment 1 of Fiser & Aslin, 2001). On each trial, a true and a foil pair was presented sequentially in the middle of the screen and participants used the left and right arrow keys for selecting the first or second pair, respectively, to indicate which pair was more familiar. Each test stimulus pair was presented for two seconds, displayed at the same size as during learning, with a one second interval between the two stimuli. The within-test trial order of the real versus foil pair was pseudo-randomly balanced across the test. Importantly, all shapes were presented an equal number of times both during the training and test. In the test, each shape appeared six times as part of a true pair, and six times as part of a foil pail. Therefore, familiarity with the individual shapes cannot be used to perform above chance on the test.

### Data analysis & measures

All data were analyzed in Python, statistics were calculated using the *scipy*, *scikit*-learn, *PyMC* (Salvatier, Wiecki, & Fonnesbeck, 2016) and *Pingouin* (Vallat, 2018) libraries. For standard statistical tests, we report the two tailed p-values. Bayes factors were calculated using the method proposed by Rouder, Speckman, Sun, Morey, & Iverson (2009) with an uninformative prior. The study was not preregistered. Trial-by-trial data supporting the main findings of this publication is available at https://osf.io/bzngs/. Code supporting the main finding and to reproduce the figures is available at github.com/jozsarato.

Eye movement data were analyzed based on whether the fixation samples were within the gaze-contingent central region of one of the cells. We focused our analysis on which cell was looked at and not the exact gaze position within cells, because stimuli were presented at full contrast and were easily identifiable from any position from within a cell. Furthermore, our hypotheses were about how observers’ gaze transitions between cells, and not about where exactly they look within cells.

On average, participants made more than seven (7.2 ± 1) transitions between the central regions of different cells in a trial. From these transition events, we calculated the proportion of looks that were performed from a shape to its pair and used this calculation for the assessment of whether the underlying statistical structure had an effect on the transitions. Proportions were used rather than the absolute number of events because the total number of transitions could also change as the learning session progressed.

Eye movement transition events were separated into two different measures that could indicate different behaviors: *exploratory transitions* and *confirmatory returns*. An exploratory transition was defined as a gaze transition to a cell for the first time during a trial, while a confirmatory return was defined as transition to a cell that had already been visited on the current trial. The difference between these two kinds of events is important because of the different types of strategies they might reflect in using predictive information. In case of an exploratory transition, the content of the next cell could be predicted/expected only if (1) the cell contained a member of a shape pair whose other member the participant already saw during the current trial, AND (2) if the participant had already learned and stored in long-term memory the spatial relationships between shapes based on the previous trials. Otherwise, the next fixation could follow only a random exploration. A significant increase from chance in the proportion of exploratory looks during the experiment that moved from one element of a true pair to the other element of the same pair would indicate evidence for learning the underlying pair structures of the scenes and the application of this knowledge in guiding eye movements.

In contrast to the exploratory case, when participants perform a confirmatory look by returning to an already visited location, they can also rely on a recent memory trace of the shape in that position in the scene in addition to the long-term learned information potentially used during an exploratory transition. This extra information can help the participants to make the “implicit hypothesis test” (i.e., to check that two shapes indeed formulate a pair, even when the long-term “hunch” of the existence of a relation between the two shapes is not sufficiently strong to drive an exploratory look). In other words, learning the identity of shape pairs can be supported either by strong enough long-term traces based on earlier experience (exploratory) or by weaker long-term memory traces but more support from very recent input (prior fixation) and direct explicit knowledge about the potential abstract structures (there must be pairs) through a confirmatory return. Comparing the separation between fractions of exploratory versus confirmatory looks throughout the experiment can indicate whether the participant relies more or less on explicit support and weak evidence indicated by the confirmatory returns.

For the analysis of temporal changes in the gaze behavior across trials, we used regression to predict the eye movement data with trial number as a predictor. We analyzed the results with two different regression methods and found that both supported the same conclusions. The first method was a simple linear regression predicting the average eye movement measures across participants (Figure 2). The second method was a linear mixed model including a random intercept for each observer that predicted a common slope for eye movements across participants (Supplementary Material S2).

To analyze how temporal changes in exploratory and confirmatory looking behavior across trials were linked to learning (Figure 3), we calculated the Pearson correlation between our eye movement measures on each trial and the performance in the final familiarity test. Next, we divided the obtained *r* values in 36 trial-long consecutive bins yielding four bins in Experiments 1 and 2 and eight bins in the twice as long Experiment 3, and analyzed with standard *t*-tests whether the *r* values in each bin were different from zero or from each other. For statistical correction of multiple comparisons, the Bonferroni method was used.

### Computational model of specific learning content

We used a model-based analysis to obtain a measure that could be fitted to all gaze transitions without relying on the selection of particular events. Our goal was to quantify how much participants' gaze trajectories changed from random exploration to a pattern determined by statistical regularities over the duration of the experiment. For each participant, the model measured the increase of alignment between looking behavior and the statistical structure of the stimuli compared to the average behavior as quantified by the distribution of transition probability across the cells of the grid. To this end, we computed the gaze transition probability matrix between the nine cells of the presentation grid for each individual participant (based on the entire experiment) and used this as the null model (essentially a Markov model). Next, for each trial, deviations from this null model were calculated based on what was presented on the screen and the gaze sequence: alpha values measure whether a presented shape increases the probability of looking next at the pair of the shape.

Because there were three types of regularities in the stimuli (link across horizontal, vertical, and diagonal orientations), the model had three parameters (α_1-3_), representing increased gaze transitions between shapes forming pairs in each of the three orientations. For example, the value of α_1_ represented an increased probability of looking from shape1, which was a member of a horizontal pair, to the position of shape2, the other shape in the pair. For each observer, the values of the three parameters were fitted trial-by-trial using the maximum likelihood method (Supplementary Material S5). To test whether these orientation-specific changes in eye movement behavior during the learning phase could predict performance in the test session, we separated the 36 test trials based on the orientation of the true pair in the trial, yielding 12 test trials for each orientation. Next, we used Pearson correlation to predict orientation specific test performance based on the fitted model parameters of each participant (Figure 4).

### Computational model of gaze fraction changes

To analyze changes in event fractions, we used Bayesian binomial regression implemented in PyMC (Salvatier et al., 2016) to model changes in the probability of within-pair eye movements as a function of trial number while being sensitive to the number of eye movements on any given trial (Figure 3C).

In the model, the likelihood was defined as follows:

$$\begin{array}{c} P\left[tr\right]\;=\;invlogit\left(\beta 0+\beta 1\;*\;tr\right) \end{array}$$

$$\begin{array}{c} k\left[tr\right]\;∼\;binomial\left(P\left[tr\right],n\left[tr\right]\right) \end{array}$$

In the above, we used standard normal priors for  *β*_0_ and *β*_1_, *tr* indicates trial number, *n[tr]* is the observed number of return eye movements on a given trial, and *k[tr]* is the observed number of within pair eye movements. We also tested a version of this model with a varying intercept for each participant, but since it yielded very similar results, we report findings for the simpler model.

### Combined model of eye movements and learning performance

We compared Experiments 1 and 2 to the second half of the Experiment 3 with a binomial regression.

*Likelihood:*

$$\begin{array}{ccc} Pcorrect\left[s\right]\; & = & \;invlogit\left(\beta 0+\beta 1*PairRate\left[s\right]\right.\\ & & \left.+\beta 2*Exp+\beta 3*\left(PairRate\left[s\right]*Exp\right)\right) \end{array}$$

$$\begin{array}{c} Ncorrect\left[s\right]\;∼\;binomial\left(Pcorrect\left[s\right],\;Ntest\right) \end{array}$$

β0-β3: had normally distributed priors with mean = 0, and SD = 2. We modeled the number of correct familiarity test responses (Ncorrect) for each subject (*s*), out of the 36 test trials (Ntest), with a binomial distribution. The probability of a correct response, was modeled as a function of within pair eye movement rate (average exploratory and confirmatory pair rate separately for each subject *s*), Experiment as a categorical predictor (Experiment 1 or Experiment 2 = 0; Experiment 3 = 1), and an interaction between Experiment and pair rate.

## Experiment 1: Explicit statistical learning influences eye movements

### Results

**Figure 1.**
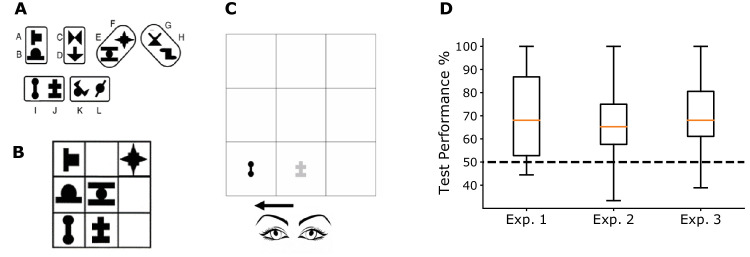
Experimental design and test results. (A) A set of 12 abstract shapes were randomly assigned to six pairs (two vertical, two horizontal, two diagonal) for each participant. (B) One example of the 144 possible scenes that were assembled from three differently oriented pairs randomly arranged on a 3 × 3 grid following the method of previous studies of spatial statistical learning. (C) Example trial snapshot of the gaze-contingent statistical learning paradigm applied in this paper with the underlying structure of the trial scene shown in B, whereas the participant's gaze moved from the bottom middle to the bottom left cell (indicated by the arrow). (D) Results of the two-interval-forced-choice familiarity test after the learning phase in the three experiments differing only in instructions and training lengths showed highly significant learning performance (N = 40, each, Error bars: full range of data). Test performance was not different across the three experiments (*F*_2,117_ = 0.89, *p* = 0.415, η_p_^2^ = 0.01).

After exploring 144 unique training scenes assembled from pairs of novel shapes for approximately 15 mins, on the two interval forced choice familiarity test, participants demonstrated significantly above chance performance (M = 70.56% CI = [64.94, 76.17], t_39_ = 7.09, *p* < 0.001, Cohen's *d* = 1.12, BF = 8.174e + 05), indicating that they acquired at least a partial knowledge about the underlying regularities (Figure 1D). Additionally, we found no significant difference between the performances with different pair orientations (F_2,78_ = 0.22, p = 0.803, η_p_^2^ = 0.006). There was no effect of orientation of the foil pairs either: participants performed equally well regardless whether the orientation of the foil and the true pairs were the same or not (*t*_39_ = 0.66, *p* = 0.514, BF = 0.209).

**Figure 2.**
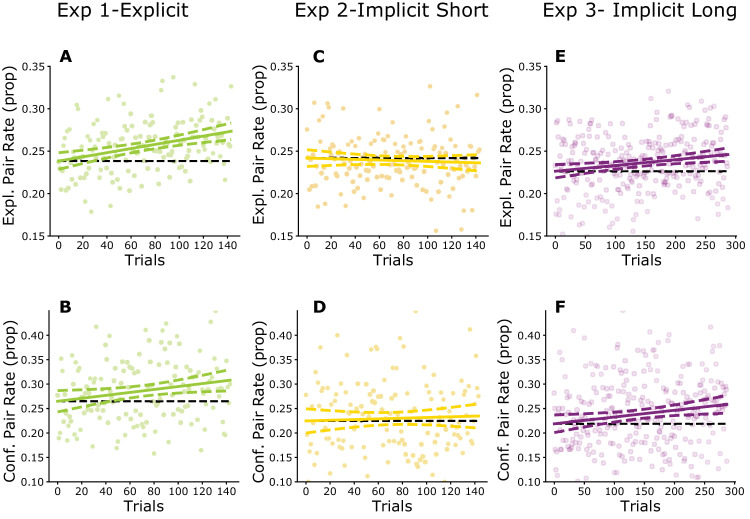
Eye movements are progressively influenced by learned statistical regularities. Columns indicate the three experiments (Experiment 1: A, B; Experiment 2: C, D; Experiment 3: E, F), rows show the two measures (exploratory and confirmatory gaze transitions) used to quantify the relation between learned underlying spatial regularities and eye movement patterns. Dots represent per trial proportion values for each observer for the two measurements, group performance is shown by the least squares regression line (solid) and the 95% confidence interval (dashed). Black dashed horizontal line indicates chance performance. Top row: The proportion of explorative eye movements (defined as first gaze visit on a trial) that were performed according to the statistical structure of the scene (moving from a shape to its pair) was increasing over-time when the instructions were explicit (Experiment 1: A, β = 0.0245, *p* < 0.001) or during long implicit learning (Experiment 3: E, β = 0.0068, *p =* *0.005*), but it stayed nonsignificant during the short implicit learning (Experiment 2: C β = −0.0039, *p =* *0.513)*. Bottom row: The same conclusions are supported by the confirmatory gaze transitions measure, the proportion of within trial returns to cells already visited on a given trial that were performed within shapes forming pairs. Again, there was a significant increase in Experiment 1 (B, β = 0.0301, *p* = 0.026, solid line) and Experiment 3 (F, β = 0.0139, *p* = 0.012), but no change in Experiment 2 (D, β = 0.007, *p =* *0.643).* The above effects are small because they include all participants, including many who did not learn. For participants who perform well on the test, the effects are considerably stronger (Supplementary Figure S8).

**Figure 3.**
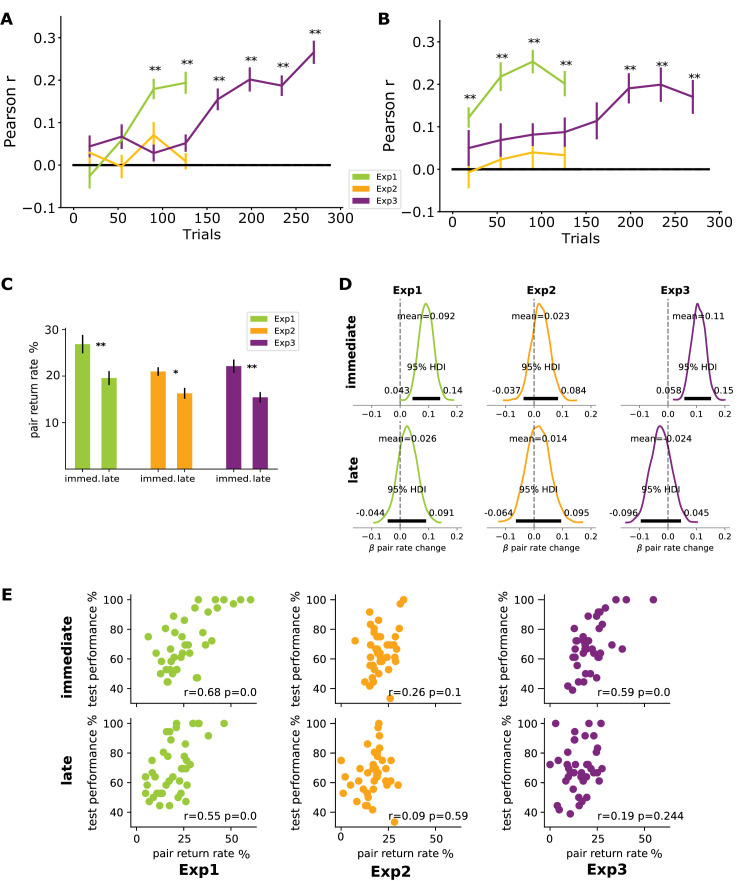
Changes in exploratory and confirmatory eye movements because of acquired knowledge about the statistical structure of the stimulus have an increasingly direct link to performance in familiarity tests. Trial-by-trial eye movement measures of each participant were correlated with individual learning success measured on the familiarity test. Single trial Pearson *r* values were averaged in successive 36-trial-long bins. (A) Within-pair exploratory gaze transition rate successfully predicted performance on the familiarity test both in Experiment 1 and Experiment 3. Exploratory looking in all three experiments was not predictive of test performance in the initial bin, but it quickly emerged to a highly predictive level in Experiment 1, unlike in Experiment 2 and in the first half of Experiment 3, where Pearson *r* values remained at chance level. However, in the second half of Experiment 3, a strong relationship between eye movements and performance emerged matching that of Experiment 1. (B) Largely the same pattern of results was found with Confirmatory as with Exploratory transitions, with a faster emergence of statistical influence only in Experiment 1. suggesting that returns could reflect a hypothesis testing process of learning. (Error bars: SEM; ** *p* < 0.01 after Bonferroni correction). (C) Separating within trial return eye movements into immediate (immed.) and late returns showed that the former were significantly performed more often within pairs in all of the three experiments. (D) Analyzing how the number of within-pair eye movements changed over the course of the experiment with a Bayesian binomial regression, showed that the fraction of within pair immediate gaze returns (top row) increased across trials in Experiments 1 and 3, whereas the fraction of within-pair later returns (bottom row) did not change in any of the three experiments. (E) predicting learning from immediate (top) and late (bottom row) return eye movements. In Experiment 1, both were predictive of test performance, whereas in Experiment 2 neither. In Experiment 3 only immediate returns had a tight relationship with learning.

Analyzing the eye movements, we first confirmed that a presented stimulus in a cell influenced the scan-path as we found that the entropy of gaze transitions was higher when a cell contained a shape rather than being empty (t_39_ = 6.48, *p* < 0.001, Cohen's *d* = 0.66, BF = 1.327e + 05, Supplementary Material S7). To investigate the effect of the learned underlying structure on eye movements, we analyzed whether the exploratory and confirmatory gaze transitions were influenced by the pair structure during training through the slope of regression (β) fitted to the proportion of exploratory and confirmatory looks across trials. The proportion of both types of gaze transitions following the pair structures was steadily increasing over the trials (Exploratory: β = 0.0245, *p* < 0.001, Figure 2A; Confirmatory: β = 0.0301, *p* = 0.026, Figure 2B). Furthermore, both measures significantly correlated with the performance on the final familiarity test (Exploratory: *r_38_* = 0.39, *p* = 0.013, BF = 3.947; Confirmatory *r_38_* = 0.70, *p* < 0.001, BF = 41040). No such correlation was found between test performance and the extent to which the scenes were explored as indicated by the number of cells visited on each trial (*r_38_* = 0.001, *p* = 0.994, BF = 0.197). This confirms that the influence of learning was specific to the spatial structure of the eye movements and not linked to some generic (e.g., attentional) changes across the experiment. Moreover, these correlations increased across the four quarters of the trials as the experiment progressed (Figure 3). Importantly, in the first half of the experiment, confirmatory gaze was significantly more predictive of test performance than exploratory gaze (Trials_1-36_: *t*_35_ = 4.43, *p* < 0.001, *BF* = 275.1; Trials_37-72_: *t*_35_ = 4.21, *p* < 0.001, *BF* = 153.1). This difference disappeared during the second half of the experiment in the last two quarters, where the learning could be predicted to a similar extent from the exploratory and confirmatory gaze selections (Trials_73-108_: *t_3_*_5_ = 2.03, *p* = 0.05, *BF* = 1.117; Trials_109-144_: t_35_ = 0.19, *p* = 0.854, *BF* = 0.182).

**Figure 4.**
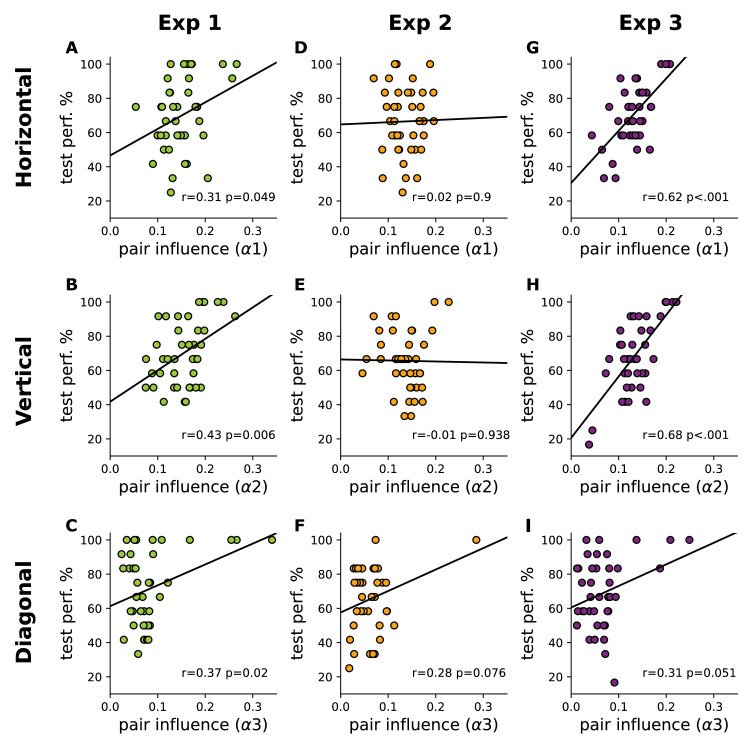
Familiarity test performance is predicted by eye movement changes because of both implicit and explicit learning of stimulus regularities. On the x axes, parameters of the model-based analysis individually fitted to all gaze-transition data are shown, indicating how strongly a particular pair structure influenced eye movements relative to the average exploration behavior of the participant. The model had three parameters, corresponding to horizontal (α_1_: top row), vertical (α_2_: middle row), and diagonal pairs (α_3_: bottom row), representing the relative increase in the number of looks that were in agreement with the spatial arrangement of the pairs. On the y axes, performance on the familiarity test trials containing true pairs from the corresponding orientation is presented. Pearson *r* and *p* and least square regression lines are shown for each condition. The specific link between eye movements and the content of learning was especially strong in Experiment 3 (right column), both for horizontal and vertical pairs. The same two directions also showed a significant relationship in Experiment 1 (left column), with a weaker relationship for diagonal pairs due to a stronger ceiling effect. None of the links were significant in Experiment 2 (middle column).

To test whether beyond the overall correlation, the specific content of learning could also be deciphered from the observer's eye movements, we correlated the orientation specific parameters (α_1-3_) of the model-based statistical analysis and the orientation specific components of learning performance based on the differently oriented pairs during the familiarity test. This test yielded clear evidence of a significant correlation between the α parameters of eye movement modulation and specific learning performance with pairs in each of three orientations (Figures 4A–C).

### Discussion

Summarizing the results of Experiment 1, we found that explicit learning of complex regularities can influence eye movement patterns at the time scale of a few dozens of minutes. Previous evidence of active sensing based on the number of fixations until finding a target (Najemnik & Geisler, 2005; Peterson & Kramer, 2001) or looking times (Hoppe & Rothkopf, 2016) have already suggested that eye movements can use environmental regularities within a single trial. Our findings extend these results to active learning by showing that, with an explicit task, the patterns of explorative eye movements become sensitive to newly learned spatial stimulus regularities that are defined across rather than within trials, and the change in eye movements during learning also reflect the amount of learning. Moreover, the explicit knowledge of the desirable structures drove the eye movements in a more “hypothesis testing” mode in the first part of learning, as an increased number of confirmatory looks were used to reinforce the accumulating knowledge matching the hypotheses. This pattern disappeared in the second half, where presumably a substantial part of the accumulated knowledge became strong enough so that exploratory looks without looking back were sufficient for interpreting the acquired information at the new fixation.

## Experiment 2–3: Implicit learning of spatial regularities

In Experiment 1, we demonstrated a direct link between learning complex regularities (the underlying visual chunks) and eye movements when an explicit instruction provided cognitive support for visual explorations of and learning from the scenes. In Experiments 2 and 3, we investigated whether this link between learning and eye movements also emerged when people were simply exposed to the stimuli without any previous knowledge or instructions about regularities within the stimuli. Since learning could only be assessed without interference with implicitness after the end of the exposure period by the familiarity test, we used two different training lengths in order to assess the link between the strength of learning and its influence on eye movements at two different stages of learning.

### Results

Participants demonstrated significant learning in the familiarity test in both experiments (Experiment 2: *t*_39_ = 6.81, *p <* *0.*001, *d* = 1.08, BF = 3.503e + 05; Experiment 3: *t*_39_ = 7.58, *p <* *0.*001, *d* = 1.2, BF = 3.504 + e06), with the performance in Experiment 3 numerically above that in Experiment 2 (Experiment 3: M = 69.65%, CI = [64.64, 74.67] vs. Experiment 2: M = 65.9%, CI = [61.38, 70.43]), but this difference was not statistically significant (*t*_78_ = 1.07, *p* = 0.286, d = 0.24, BF = 0.38) (Figure 1D). To confirm that the gaze contingent paradigm did not interfere with statistical learning and thus the observed learning effects were not particular to the experiment, we confirmed that familiarity test performance was in line with the 68–78% correct reported in previous studies on visual statistical learning (Fiser & Aslin, 2001; Fiser & Aslin, 2005; Turk-Browne et al., 2005). Similarly to Experiment 1, we found no difference between the test performances with different orientations in Experiment 2 (*F*_2,78_ = 0.53, *p* = 0.589, η_p_^2^ = 0.013) or in Experiment 3 (*F*_2,78_ = 2.16, *p* = 0.122, η_p_^2^ = 0.052). In addition, there was no effect of whether the orientation of the foil and the true pair matched or not (Experiment 2: *t*_39_ = 1.3, *p* = 0.197, BF = 0.378; Experiment 3: *t*_39_ = 1.26, *p* = 0.216, BF = 0.354).

Turning to eye movements, we first analyzed gaze transition entropy (Supplementary Material S7) and confirmed that gaze direction was more unpredictable when initiated from cells containing a shape than from empty cells both in Experiment 2 (t_39_ = 6.66, *p* < 0.001, Cohen's *d* = 0.66, BF = 2.277e + 05) and Experiment 3 (t_39_ = 6.33, *p* < 0.001, Cohen's *d* = 0.34, BF = 8.535e+04). Analyzing the effect of the underlying structure on the eye movements with least-square regression, we found a striking contrast between the two experiments. In Experiment 2, we found no evidence conveyed by regression slopes of any increase in within-pair fixations rates either for exploratory (*β* = −0.0039, *p* = 0.513) or for confirmatory looks (*β* = 0.007, *p* = 0.643) (Figures 2C and 2D). In contrast, and more similarly to Experiment 1, observers’ changing fixation rates in Experiment 3 reflected an increasing influence of the pair structure on eye movements over time both in exploratory (*β* = 0.0068, *p* = 0.005) and confirmatory looks (*β* = 0.0139, *p* = 0.012) (Figures 2E and 2F). Compensating the potential confounding effect of variable numbers of eye movements within trials, we reanalyzed the data with a Bayesian mixed model and confirmed the significance of the regression slope in Experiment 3, and the lack of such effect in Experiment 2 (Supplementary Material S2).

### Eye movements predict implicit learning performance

In Experiment 2, the eye movement measures were not predictive of the outcome of the familiarity test (Exploratory: *r*_38_ = 0.17, *p* = 0.308, BF = 0.325; Confirmatory *r*_38_ = 0.18, *p =* *0.*26, BF = 0.363). In contrast, in Experiment 3, both measures had a strong correlation with learning performance (Exploratory: *r*_38_ = 0.55, *p <* *0.*001, BF = 138.5; Confirmatory *r*_38_ = 0.54, *p* < 0.001, BF = 110.3). We also confirmed that these correlational findings in Experiment 3 do not depend on a few high performing outliers (Supplementary Material S1). This relationship between learning and eye movements in Experiment 3 emerged gradually and revealed the strong link only by the second half of the experiment (Figures 3A and 3B, see also Supplementary Material S3). Similarly to the control test run in Experiment 1, a general measure based on the overall exploration of scenes as quantified by the average number of cells visited was not predictive of test-performance in either of the implicit experiments (Experiment 2: *r*_38_ = −0.18, *p* = 0.268 BF = 0.356; Experiment 3: *r*_38_ = 0.28, *p* = 0.082, BF = 0.845), excluding the possibility that simple motivational effects could be responsible for our findings.

In contrast to the congruence between Experiments 1 and 3 in terms of the general link between learning and eye movements, the more refined comparison between confirmatory and exploratory looks showed a pattern in Experiment 3 markedly different from that found in Experiment 1. We found no difference between the extent to which exploratory and confirmatory gaze predicted learning in any of the 36-trial-bins of Experiment 2 or Experiment 3 (Supplementary Material S6). This suggests that, unlike in Experiment 1, participants in Experiment 3 did not rely on a notably stronger cognitive support for clearly formulated “hypotheses” about the structure of the scenes at any point during the implicit experiments that would be indicated by increased effect of confirmatory returns.

To support this conclusion, we sought for independent evidence that confirmatory looks indeed contributed to learning in a “hypothesis-testing” manner. It is clear that any returning fixation to the same shape in a scene could be either accidental or causal, where causality means that a weaker memory trace is augmented by a second sensory input for evaluating a direct hypothesis. However, if shapes A and B belong to the same pair, an immediate return (fixate on A, next on B, then again on A) would be more in line with the concept of evaluating a direct hypothesis than a late return after more than one intermediate fixations (e. g., A-D-E-B-A). Based on this reasoning, we compared three aspects of the immediate versus late confirmatory returns: their fraction occurring within pairs as opposed to between pairs, the change of this fraction as time passes during exposure and the power of the immediate versus later returns to predict learning performance. The fraction of immediate returns occurring within pairs in all three experiments were significantly higher than those in late returns (Exp1: *t*_39_ = 4.87, *p* < 0.001, BF = 1128.8; Exp2: *t*_39_ = 3.25, *p* = 0.002, BF = 14.15; Experiment 3: *t*_39_ = 4.67, *p* < 0.001, BF = 627.48; Figure 3C). With the passage of time during exposure, this fraction increased for immediate returns in Experiments 1 & 3 (Experiment 1: β_HDI95_ = 0.043–0.14; Experiment 2: β_HDI95_ = −0.0.37 to 0.84; Experiment 3: β_HDI95_ = 0.058–0.1, Figure 3D) but did not change for later returns in any of the experiments (Experiment 1: β_HDI95_ = −0.044 to 0.091; Experiment 2: β_HDI95_ = −0.064 to 0.095; Experiment 3: β_HDI95_ = −0.06 to 0.045). These findings support the idea that immediate returns are confirming implicit hypotheses based on the accumulating knowledge while a larger fraction of later returns are more accidental and driven less by the emerging knowledge related to pairs.

A closer look at gaze returns’ predictive power on learning provided further support for this idea (Figure 3E). In the case of the short implicit Experiment 2, neither type of return had significant predictive power on learning performance (immediate: *r*_38_ = 0.26, *p* = 0.103, BF = 0.713; late: *r*_38_ = 0.088, *p* = 0.59, BF = 0.227). On the other hand, returns in both Experiments 1 (explicit) and 3 (long implicit) had significant predicting power on learning but with different patterns. In Experiment 3, only immediate returns had significant relations to learning performance (*r*_38_ = 0.59, *p* = 0.000052, BF = 529.8) while late returns did not (*r*_38_ = 0.19, *p* = 0.244, BF = 0.379). In contrast, in Experiment 1, both immediate (*r*_38_ = 0.68, *p* < 0.001, BF = 1187) and late (*r*_38_ = 0.55, *p* < 0.001, BF = 120.5) returns had significant correlation with learning suggesting that explicit top-down cognitive knowledge factored in eye movements more prominently in Experiment 1 than in Experiment 3. Together, these results strongly suggest that confirmatory looks and especially immediate confirmatory looks are indicators of participants' learning being driven by implicit or explicit “hypothesis-testing” strategies in which top-down candidate hypotheses are evaluated.

Detailed analysis of the link between the orientation-specific changes of eye movements (model α_1-3_) and familiarity test performance also revealed a strong difference between the short and long implicit experiments. Predictive relationships were absent in Experiment 2 (α_1_: *r*_38_ = 0.02 *p* = 0.9, BF = 0.198; α_2_ *r*_38_ = −0.01, *p* = 0.938, BF = 0.198; α_3_: *r*_38_ = 0.28, *p* = 0.076, BF = 0.904; Figures 4D through 4F), whereas in Experiment 3, there was a very strong relationship between the magnitude of orientation-specific influence on observer's eye movements and their pair-specific test performance. For both horizontal and vertical pairs, this effect was highly significant (α_1_: *r*_38_ = 0.62, *p* < 0.001, BF = 1442.37; α_2_ *r*_38_ = 0.68, *p* < 0.001, BF = 13370; Figures 4 G and 4H), while for diagonal pairs, it was weaker and marginally significant (*r*_38_ = 0.31, *p = 0.*051*, BF =* 1.241*,* Figure 4I). Using a resampling based approach(Simon & Bruce, 1991), we confirmed that these correlations in Experiment 3 were not due to general learning effects, but they were highly specific to the particular features the participants learned (Supplementary Material S4).

## Experiments 1 to 3: The relationship between learning and eye movements

Although our results so far demonstrate that learning both explicitly and implicitly changes eye movement patterns, the extent to which the resulting effects are similar across the two conditions is unclear. It is obvious that eye movements in Experiment 2 and the first half of Experiment 3 were similar (Figure 3, S1 & S3). However, it is less clear whether the changes in eye movement patterns during the second half of Experiment 3, when participants had already gained some implicit knowledge of the structure of the input, are comparable to the changes due to the gain from explicit instructions in Experiment 1.

To settle this question, we performed four binomial regression analyses. Binomial regression allows modeling the probability of a correct response taking into account the number of trials, and we used it here to test if the relationship between eye movements and test performance is similar across experiments. To this end, we used three predictors: (1) exploratory or confirmatory gaze; (2) experiment as a categorical variable; and (3) an interaction term between these two—the main focus of this analysis. We used these variables to predict test performance in Experiment 3 combined with Experiment 1 or Experiment 2 in separate analyses.

**Figure 5.**
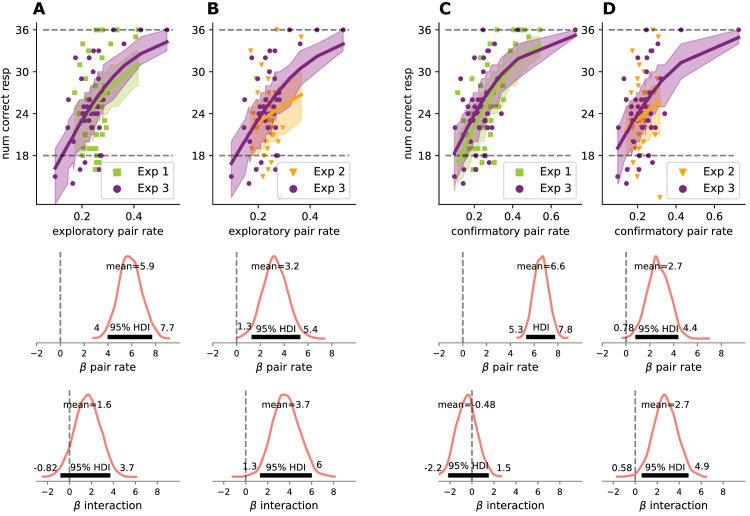
The relationship between eye movements and learning across experiments. Top panels: data with the posterior predictive distribution of four separate Bayesian binomial regression models. Each data point represents one participant, colored lines represent the mean posterior predictive, dashed lines at 18 and 36 indicate chance and perfect performance, respectively. Mid-panels: distribution of possible parameter values for the effect of within pair eye movements in predicting familiarity test performance, for exploratory (A, B) and confirmatory (C, D) looks. Bottom panels: Distribution of possible parameter values for the interaction term between experiment and eye movements with the 95% highest posterior density interval (HDI). (A) Comparison of slopes for exploratory looks and test performance in Experiment 1 and Experiment 3 indicated no interaction. (B) Comparison of slopes for exploratory looks and test performance in Experiment 2 and Experiment 3 revealed a significant interaction, with a stronger relationship in Experiment 3. (C, D) The same analysis for confirmatory eye movements as in A and B resulted in the same pattern of performance.

Comparing Experiment 1 and Experiment 3 in the same model, we found a highly significant effect of exploratory eye movements on the prediction of learning (HDI95% = 4–7.8; Figure 5A middle row) and no interaction between the experiments (HDI95% = −0.74–4.1; Figure 5A bottom row). The same analysis comparing Experiment 2 and Experiment 3 (Figure 5B) showed a strong effect of exploratory eye movements on the prediction of learning (HDI95% = 1.3–5.4), but this influence also had a significant interaction with experiment category (HDI95% = 1.4–6). The same pattern was replicated with confirmatory eye movements (Figures 5C and 5D): in the combined analysis of Experiments 1 and 3, gaze was a strong predictor of learning (HDI95% = 5.3–7.8; Figure 5C, middle row), without interaction with experiment type (HDI95% = −2.3–1.2; Figure 5C, bottom row), while confirmatory eye movements in Experiments 2 and 3 were significant predictors of learning (HDI95% = 0.88–4.6; Figure 5D middle row), but with a clear interaction with experiment category (HDI95% = 0.59–5.1; Figure 5D bottom row).

These analyses indicate that although the (relatively weak) relationship between eye movements and learning was similar across Experiments 2 and 3, as implicit knowledge accumulated further in Experiment 3, it started to influence eye movements more strongly, and the eye movement patterns in Experiment 3 were influenced in the same way as in the explicit instruction condition of Experiment 1. Meanwhile, this pattern was significantly different from those in Experiment 2. Thus these results confirm our hypothesis that in our experiments, the amount of the acquired knowledge is the main driving force behind the changes in eye movement patterns regardless of the explicit or implicit nature of the experimental conditions. In other words, the influence of learned knowledge of environmental statistics on eye movements is automatic, and it does not require a well-defined task to emerge. However, we also found that although this effect was tightly linked to the specific knowledge acquired about the statistics of the input, a comparably large learning in the familiarity test of Experiment 2 emerged without any detectable influence of this learning on eye movements. This apparent contradiction will be discussed in context below.

## General discussion

Using a novel gaze-contingent statistical learning paradigm in our study, we clarified five aspects of how sensory learning and eye movement patterns interact. First, we confirmed that knowledge acquired through statistical learning about the underlying structure of the visual environment that is learnable only across several scenes has an effect on the patterns of eye movements even within the short time period of a single experiment as required by active learning. Second, we showed that this effect on eye movements emerges gradually as knowledge about the structure is accumulated and it is highly specific to the learned structural composition of the incoming sensory input. This is indicated by the individual looking patterns reliably reflecting participant's specific knowledge about the orientations of the underlying chunks in the current scene. Third, we clarified that significant knowledge of the underlying statistical regularities of the visual scenes can emerge before any detectable influence on eye movements, possibly because eye movements and the familiarity test are sensitive to different types of knowledge. Fourth, we found that the characteristics of the eye movements leading to knowledge accumulation in a set of unknown visual scenes are substantially different during the early stage of explicit and implicit learning as indicated by the different proportion of exploratory and confirmatory looks during the first part of the learning session in the two conditions. Fifth, we demonstrated that despite this early difference, once sufficient knowledge is gathered through implicit learning for a prolonged time, the general pattern of interaction between knowledge and eye movements becomes very similar under the explicit and implicit conditions. Below, we provide an expanded discussion of these findings.

In empirical investigations of the relationship between eye movements and available knowledge, the latter can include information collected at three different and roughly separable time scales: very long-term consolidated knowledge, recent knowledge collected in the past minutes to hours by viewing previous scenes within a study, and current knowledge gathered by multiple fixations from the presently displayed scene. A large fraction of previous studies explored the relationship between eye movements and explicitly or implicitly evoked internal knowledge at the longest and shortest scales (knowledge-based eye movement guidance and active sensing) (Hoppe & Rothkopf, 2019; Morvan & Maloney, 2012; Najemnik & Geisler, 2005; Peterson & Kramer, 2001; Yang, Lengyel, & Wolpert, 2017). Although these studies provided several important observations about how internal knowledge influences eye movements, they did not address the first main question of active learning: Does new information about the underlying structure of the sensory input get incorporated into the existing knowledge about the environment and start modulating eye movements immediately?

Studies investigating the effect of learning on eye movements at the intermediate “recent” scale that focused on learning spatial regularities (Boettcher, et al., 2022; Brockmole & Henderson, 2006; Chun & Jiang, 1998; Geng & Behrmann, 2005; Jiang, 2018; Jones & Kaschak, 2012) rather than on simple temporal ones (Glimcher, 2003; Hoppe & Rothkopf, 2016) confirmed that humans learn implicitly the link between target location and the structure of the underlying background. These studies used either only the first fixation (Jiang et al., 2014; Jones & Kaschak, 2012) or a measure of the efficiency of the fixation pattern converging toward the target (Brockmole & Henderson, 2006; Chukoskie, Snider, Mozer, Krauzlis, & Sejnowski, 2013) as an indicator of attentional selection. Thus these studies do address the first question of active learning as they demonstrate the incorporation of recent stimulus statistics into long-term memory, albeit focusing only on the probabilistic relation between target position and background identity within a scene. However, they evaluated the effect of this learning still only within the context of a specific task. Therefore these studies cannot adequately address the second main question of active learning: Is the interaction between newly acquired knowledge and eye movement in humans occurring perpetually even without executing any explicit cognitive task?

Our study differs from these previous approaches on three counts. First, the relevant spatiotemporal statistics of the environment the observers need to learn are more complex than those in earlier studies, and they are more reminiscent of natural learning conditions because the pairs need to be learned and identified across multiple scenes in various contexts. Second, the eye movements are not constrained by any specific task (especially in Experiments 2 and 3, but even in Experiment 1 the task is far less well defined than a visual target search), and their modulation by internal knowledge is much more specific than a simple preferential selection of a quadrant of the screen. Third, through the continuous measurement of exploratory and confirmatory looks, we could track the evolution of different aspects of the emerging interplay between learning and eye movement patterns at high resolution not measured before. Our results based on these advances showing a tight link between eye movements and dynamically changing internal knowledge therefore provide the most direct evidence to date that humans follow an active learning strategy during exploration of an unknown environment.

Because of two aspects of our study, our results are in line with but can be interpreted significantly differently than the concept behind the “attention selection” framework (Theeuwes, 2019) and its extension to statistical learning (Theeuwes et al., 2022) ​​proposed recently. First, the lack of any well-defined goal in our paradigm and the complexity of the relevant statistically learned information at any one moment makes it hard to justify the existence of a central saliency map that combines goals and input with all memory-based biases to serve attentional processes unless attention and saliency map are defined very broadly as any bias and any restrictive selection that happens anywhere in the processing stream. Second and more importantly, attentional selection is a unidirectional framework modeling how sensory and internal factors influence attention, whereas both our study and the concept of active learning in general focus on the closed-loop bidirectional interaction of how internal knowledge shapes the way new information is acquired and in turn, how this biased information determines further what will be selected, experienced, and learned next. This view and our results are better accommodated by the “premembering experience” functional account of memory proposed recently (Nobre & Stokes, 2019). This mechanism has been termed as “proactive attention,” but it is essentially the process of goal-oriented sensory information processing itself with a continuous interplay between new sensory input and top-down memory-related control (Chun & Turk-Browne, 2007; Gottlieb, 2012). Computationally, this mechanism can be well described as a hierarchical probabilistic processing of information (Lake, Salakhutdinov, & Tenenbaum, 2015) that has been recently proposed to capture the interaction between perception and learning (Fiser & Lengyel, 2019; Fiser & Lengyel, 2022).

The similarity of overall performances in the three experiments raises the question: If the accuracy in Experiment 2 is almost as good as in the other two experiments but without any noticeable scene-related eye movement changes, what can be said about the exact role of eye movements during statistical learning? The first possible explanation of this result is that our measurement is not sensitive enough to pick up the changes in the pattern of eye movement that are already manifested even after a shorter learning phase. However, this is unlikely because the correlation between familiarity and the eye movement patterns remained insignificantly low throughout both Experiment 2 and the first part of Experiment 3, whereas it increased drastically in the second part, suggesting that regardless of the actual performance in the familiarity test, the eye movements started to align notably with the acquired knowledge only in the second half of Experiment 3.

The more intriguing alternative explanation is that learning and restructuring of internal knowledge occurs throughout the entire period of practice, but the 2-AFC familiarity measure is unsuited for tracking the full complexity of the developing underlying representation. Later, in the second half of Experiment 3, after experiencing the same two shapes in a given arrangement multiple additional times, observers might encode the actual spatial layout of the pair more strongly in the internal representation beyond the identity of the shapes and their connectedness. This additional information might not necessarily improve the 2-AFC results, but the knowledge about these pairs becomes richer and more specific and can engage with eye movements during exploration. Although this explanation is speculative at this point, there are examples of internal representations transforming during extended exposure (Goujon, Didierjean, & Poulet, 2014; Kellman & Garrigan, 2009; Sun, Merrill, & Peterson, 2001). Moreover, this explanation offers a direct prediction: An appropriate measure of confidence or knowledge about the layout of the pairs would detect a significant difference between performances in the short and long version of the experiment. Importantly, the lack of significant differences between the familiarity results of the three experiments in no way influences the final conclusions of the present work, which are concerned with the existence and specificity of the relationship between changes in the eye movement patterns and newly learned structures of the underlying visual scenes in general.

We found two notable differences between the results of eye movements with and without the explicit task. First, changes in eye movement patterns occurred much faster in the explicit case, second, in the first half of the explicit task condition, the fraction of confirmatory looks following the patterns of the underlying shape pairs increased much faster than the fraction within exploratory looks, while in the implicit conditions the two fractions changed in close agreement with each other across the entire experiment. We interpreted these results by suggesting that the explicit knowledge of what structures might make up the scenes enabled a “hypothesis testing” (Friston, Adams, Perrinet, 2012; Gregory, 1980) type of learning at the beginning of the experiment, where the participants consciously or unconsciously evaluated whether particular pairs of shapes complied with a prespecified rule. Once the accumulated knowledge in the first half of Experiment 1 became substantial enough to evoke sufficient information by a single glimpse at a shape so that exploratory looks without looking back sufficed to interpret the acquired information at the second fixation, the fractions of the two types of looks converged and progressed jointly. In contrast, during implicit learning those fractions never diverged from each other, perhaps indicating the “modus operandi” for active learning in situations without strong internal knowledge about the structure of the underlying scene or without a strong task-related influence imposed on eye movements. Again, this account is speculative but provides a testable hypothesis: If instead of the beginning of the experiment, explicit information relevant to the spatial layout is provided at some intermediate point during the course of learning or conversely, an earlier acquired information is explicitly revoked at some point, these modifications should be reflected through the changes in the relative ratios of the exploratory and confirmatory looks.

Our study was designed to investigate the effect of implicit versus explicit learning. However, there are three separable aspects in any behavioral experimental setup where the implicit-explicit distinction can be defined: the task used for acquiring new knowledge can be implicit or explicit, the task could allow the observer to gain in parallel both implicit and explicit knowledge about the underlying structure of the environment, and the test in which this knowledge is utilized can be defined as asking the participant for a nonspecific implicit impression or for completing an explicit task. The implicit or explicit nature of any of these aspects can result in very different behavioral patterns (Castro-Rodrigues et al., 2022) and a complete separation of the effects of even one aspect, explicit vs. implicit internal representations, is virtually impossible, as it depends on the exact measure of learning and the definitions of implicitness (Batterink, Paller, & Reber, 2019; Christiansen, 2019; Ericsson & Simon, 1980; Nisbett & Wilson, 1977). In our experimental design, we explored the effect of both implicit and explicit learning tasks, as a result (Castro-Rodrigues et al., 2022), observers acquired individually different mixes of implicit and explicit knowledge and were tested in a nonspecific implicit test condition. Under these circumstances, we found that although the training task influenced eye movements differentially (see above), after observers were exposed to the input structure for a prolonged time, the distinctions in the general pattern of interaction between acquired knowledge and eye movements under the explicit and implicit conditions disappeared (Figure 5, Supplementary Figure S1). Although this suggests that during active learning of unknown statistical scenes the pattern of eye movements increasingly comply with the underlying structure of the scenes regardless of how the necessary knowledge is gathered, further studies are required to settle the universality and scope of this observation.

## Conclusions

In conclusion, we provided evidence that after sufficient learning, a tight link emerges between human visual information sampling strategies manifested by eye movements and the emerging internal knowledge of environmental regularities. Although observers can collect a considerable amount of information by implicit statistical learning without any noticeable gaze pattern change, learning without a task will nevertheless at some point produce the same kind of effects on eye movements as the explicit version of the task does. Our results frame natural vision as a process, in which active selection from the incoming information and internal knowledge jointly determine both the interpretation of the input and further changes in the internal knowledge, much in the same way as predicted by active learning.

## Supplementary Material

Supplement 1
