# Supplementary material for: Eye movements reflect active statistical learning
Source: J Vis. 2024 May 31;24(5):17. doi: 10.1167/jov.24.5.17 (PMC11146064; doi:10.1167/jov.24.5.17)
Supplement: Supplement 1 [file jovi-24-5-17_s001.pdf]

Eye movements reflect active statistical learning Supplementary Material (S1-S9)

S1: Comparing pair transition measures across explicit and implicit task conditions

We assessed whether the effect of stimulus statistics on eye-movements during implicit learning became similar to that measured in the task with explicit instructions. To this end, we compared the influence of statistical structure on eye-movements in Explicit Task condition (Exp 1) with the two Implicit Task conditions (Exps 2,3), where the Long Implicit task condition (Exp 3) was split into first and second halves. Since Exp 2 and the 1st half of Exp 3 showed identical results (Exploratory: t(78)=1.509 p=.1352 BF=0.622 , Confirmatory: t(78)=0.064, p=.9494, BF=0.233), we focused on comparing Exps 1 and 3. Results were analyzed in two ways, by combining all the data and by separating participants into groups based on test performance.

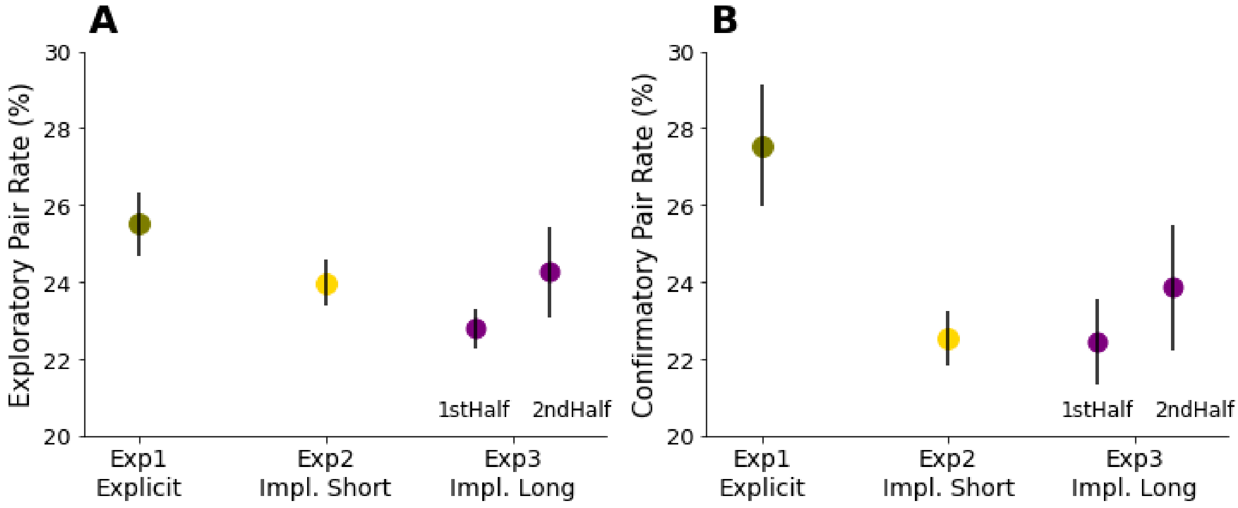

FigS1.1: **Pair transition measures across experiments**. Average performance of the two main pair transition measures (**A:** Exploratory, **B:** Confirmatory) in the three experiments, splitting Exp 3 into two half periods . (X-axis: experiments, Y-axis: proportion of within pair transitions, Error bars: SEM)

The comparisons of average performances of all participants combined are shown in Figure S1.1 with the results of statistical analyses in Table S1. The influence of the statistical structure on eye-movements in the Long Implicit experiment (Exp 3) during the first half of learning was below that found in the Explicit experiment (Exp 1). This observation was confirmed by the computed Bayes factors substantially favoring the hypothesis that the two measures indicate effects of different magnitudes in both Confirmatory and Exploratory cases (Table S1). In contrast, both measures increased in the second half of Exp 3, making the effects of the Long implicit experiment more similar to those in the Explicit experiment. Accordingly, there was no significant difference between either of the two measures when this second half of Exp 3 was compared to Exp 1. The Bayes factors obtained for this second half of Exp 3 supported substantially the hypothesis that there was, indeed, no difference between the Long Implicit and Explicit conditions for the Explorative looks (Bayes Factor = 0.325). For Confirmatory looks, which

were substantially higher during Explicit learning, the resulting Bayes Factor of 0.727 was indecisive, it did not support either of the two hypotheses.

**Measure**	Exp 1 *vs.* Exp 3 1st Half	Exp 1 vs. Exp 3 2nd Half
Exploratory Pair Rate	*t(78)*=2.83, *p*=.006, BF=6.86	*t(78)*=0.88, *p*= .382, BF=0.325
Confirmatory Pair Rate	*t(78)*=2.61, *p*=.011, BF=4.21	*t(78)*=1.63,*p*=.108, BF=0.727

Table S1: Similarity in pair transition measures across Experiments 1 and 3 as measured by t-test and Bayes Factor analysis for the two main pair transition measures Confirmatory (Row 1) and Exploratory (Row 2) using data from either the first or the second half of Exp 3.

A more detailed, albeit qualitative inspection of the overall tendencies is possible when the participants are binned according to their learning performance, and the data is combined for all participants (**FigS1.2A-C),** or separated for different types of pair stimuli (Horizontal, Vertical, Diagonal) (**FigS1.2D-E**). Since such a binning and separation of results limits the sample sizes in each condition, the data do not have sufficient statistical power for making comparisons for each learning group. Nevertheless, it is clearly observable in almost all the different panels that by the second half of Exp 3, good learners (even outside the 100% performance bin) approach or even exceed the amount of statistical influence reflected in their eye-movements compared to those of the explicit learners in Exp 1. The sole exception is Confirmatory looks, where only learners in the 100% bin of Exp 3 demonstrated a statistical influence that was arguably comparable to those in Exp 1 (**FigS1.2 B)**.

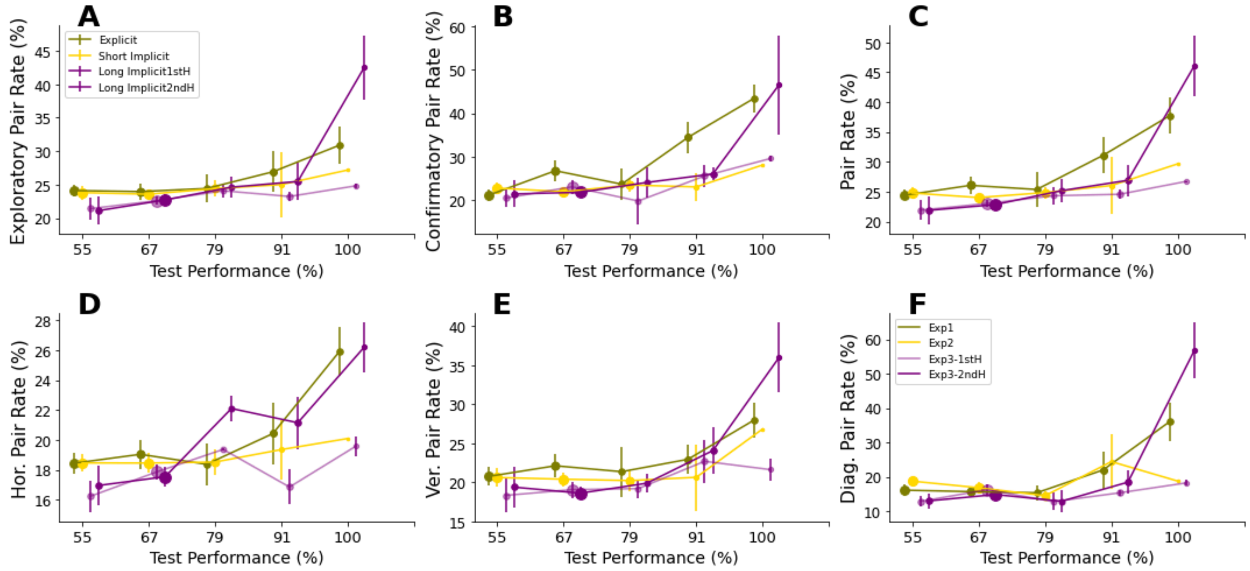

FigS1.2: **Pair transition measures separated by learning performance and pair orientation.** In all panels, participants were binned by test performance into 5 bins between 50 and 100% performance indicated by the mean of the bins on the x-axis. The last bin included only the 100% learners. The colors indicate the three different experiments (see legend in panel A) with Exp 3 separated into two halves, denoted by different shades of purple. *Top Row:* Overall pair-rate measures (A: Exploratory, B: Confirmatory, C: All eye-movements combined across Exploratory and Confirmatory). *Bottom Row:* Direction-specific pair-rates (D: Horizontal, E: Vertical, F: Diagonal). The

x position of the data symbols was jittered around the mean test performance in each bin for easier visibility. The size of the dot symbols is proportional to the number of participants in each bin.

The reported relationship between learning and eye-movements was not the result of only a few 100% learners. All of our comparative effects in Exp 1 and Exp 3 remained the same even when the best performers were not included in the analysis. In Exp 1, Exploratory eye-movements had a significant relationship with learning, only if 100% performers were included (everyone: p=.013 *vs.* excluding 100%: p=.193). In Exp 3, while there was no relationship in the first half (everyone: p=.061 *vs* excluding 100%: p= .147), a significant relationship emerged in the second half of the experiment irrespective of the inclusion of 100% learners (all participants: p<.001; excluding 100% performers: p=.021). The pattern was similar for Confirmatory looks, where the relationship was very strong in Exp 1 (all participants: p<.001; excluding 100% performers: p<.001), while the effect was weaker, but also significant in the second half of Exp 3 (all: p<.001, excluding 100% p=.019).

We did not remove outliers in our datasets not only based on the concept of keeping the intactness of the collected data but also for two other reasons. First, in Figure 4, an outlier in one of the eye movement measures (eg: diagonal gaze) was not an outlier in several other measures, therefore, there was no valid basis for such a trimming. Second, focusing on our main dependent variable -test performance- instead of eye movements when checking for outliers, we found no outliers in test performance in any of the three experiments when using the conventional criterion of the data point residing outside +/- 2.5 SD from the mean. Besides these factors, we also show in Figure S1.2 (grouping participants by learning performance) that while the strongest eye movements effects were shown by the best learners, these effects were also present by less well performing learners. Therefore, we are confident in saying that our main results do not depend on a few participants' performance.

S2: Bayesian analysis of eye-movement changes

The linear regression analysis applied for obtaining **Fig 2** in the main text is sensitive only to the overall proportion of within-pair transitions, but not to the number of eye-movements performed in a trial. This could make the result potentially more noisy and sensitive to trials with only a few eye-movements (for example, when a 100% or 0% within-pair count can occur by chance). To control for this caveat, we modeled the number of within-pair transitions, together with the number of gaze transitions the participants actually performed on each trial with the following Bayesian mixed model:

Likelihood:

$$Ktr \sim binomial(Ptr,\ Ntr)$$
$$Ptr = invlogit(\beta 0[subj] + \beta 1 * TrNum\ )$$

Priors:

$$\beta 1 = Normal(0,.1)$$
$$\beta 0[subj] = Normal(B0mean, B0sd)$$
$$\beta 0mean = Normal(0, 1)$$
$$\beta 0sd = HalfNormal(1)$$

In the model, a common slope ($\beta1$) was used to capture the change in within-pair transitions, while the intercept was independent for each participant ($\beta0subj$). Parameters $\beta0subj$ and $\beta1$were used in a linear model to predict the probability of within-pair transitions on each trial (Ptr). The number of within-pair transitions (K_{tr}) on a trial was predicted from the number of transitions (Ntr) and Ptr using a binomial distribution. This analysis explored the extent to which this binomial probability changed as a function of trial number. The posterior probabilities for parameters $\beta0[subj], \beta1, mu, sd$ were estimated for Exps 1-3 separately, using PyMC3 (Salvatier et al, 2016) and NUTS with 1000 tuning and 1000 inference samples.

The results of this analysis confirmed the observations of the main text: there was a significant increase in within-pair looks across trials in Exp 1 and Exp 3, as 90% credible intervals did not contain 0, and no change in Exp 2 since its credible interval was centered around 0 (**Fig S2B**). The overall change in the number of looks was very similar during the course of the experiments in Exp 1 and 3 as indicated by the highly overlapping 90% credible intervals (**Fig S2C**).

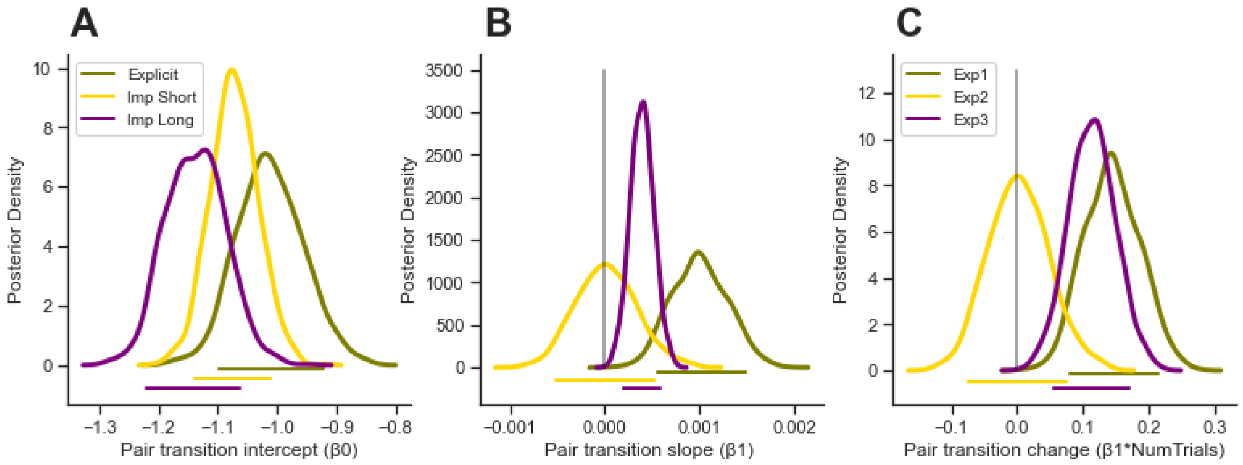

FigS2: Pair transition rate across trials: Bayesian mixed model results with the mean posterior probability distribution for Exps 1-3 (see color in legend in A) and the 90% credible interval marked at the bottom of each panel with a horizontal line . **A)** Mean posterior probabilities for the intercept $B0$. **B)** Mean posterior probabilities for the slope$\beta1$ Exps 1-3. Gray vertical line marks zero slope, i.e. no change, which is below the credible intervals for Exp 1 & 3 but not for Exp 2. The lower but more certain values for Exp 3 is explained by the fact that the pair transition rate reaches a similar level as in Exp 1, but over the course of twice as many trials. **C)** Posterior predictive distribution of change in pair transition rate throughout the total duration of the experiments (Slope □1* number of trials) was clearly above zero and very similar in Exp 1 and Exp 3, but not different from zero in Exp 1.

S3: Pair Transition Rates over-time

In order to gain information about the emergence of relationships between eye-movements and learning, we calculated a moving average of the relationship across 36 binned trials including only those participants, who demonstrated some learning, that is whose test performance was above 60% in the familiarity test (**FigS3)**.

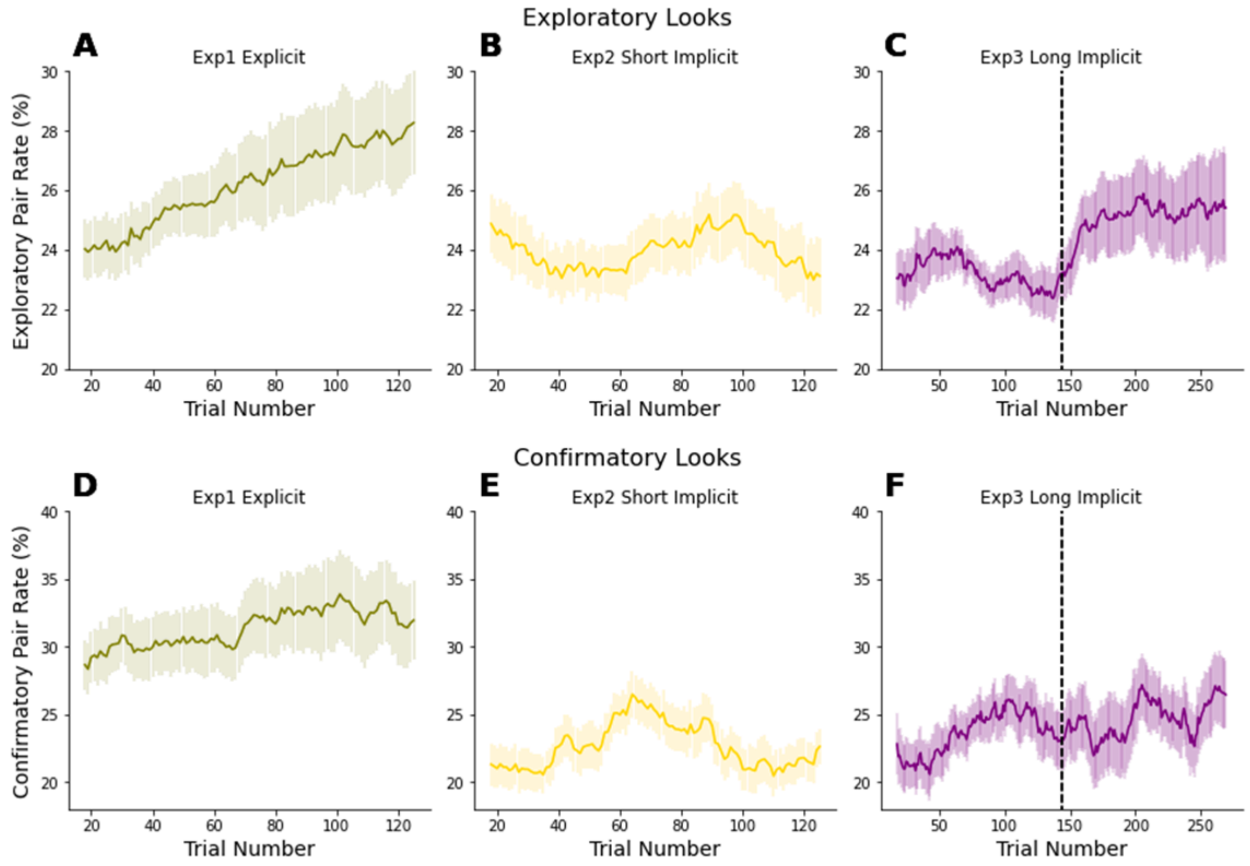

FigS3: Moving average of within-pair transitions. Changes are presented for all within-pair transitions for exploratory (top-row) and confirmatory (bottom-row) eye- movements. Error bars indicate SEM. Note, different y-ranges for the two rows. In **E,F** dashed vertical lines indicate the half of the Exp 3, that is the total length of Exp 1-2.

For Exploratory looks, Exp 1 shows a steady increase of the effect across trials in contrast to Exp 2, where locally averaged looks remain the same across the experiment. The first half of Exp 3 displays a very similar pattern to that in Exp 2, but in around the transition to the second half, a rapid increase in the magnitude of the effect emerges, and a slower but monotonic increase remains until the end of the experiment. The explicit knowledge provided in Exp 1 generated a slightly different overall pattern for Confirmatory looks. In Exp 1 the effect was already prominent in the first bin with a more modest improvement from until the end of the experiment. The similarity between Exp 2 and the first half of Exp 3 remained together with the nonsignificant changes in this period, but the increase in the second half of Experiment 3 was less pronounced.

S4: Analysis of the Specificity of Correlations

In the main text, we found strong correlations between $\alpha_{1\text{-}3}$ and the corresponding direction specific performance (**Fig 4**). However, the specificity of these correlations could be questioned as they might indicate just a general effect of learning, with the same participants performing similarly well at the

different orientations. To confirm the true specificity of these correlations, one needs to demonstrate that, for example, the correlation between α_1 (measuring horizontal statistical influence in eye-movements) and the corresponding test performance of familiarity with horizontal stimuli is, in fact, significantly stronger than what would be obtained by correlating α_1 with the non-corresponding vertical or diagonal performance. To test whether this is the case, we used a permutation-based approach, where (after z-scoring the values) for each participant, we randomly shuffled α_{1-3} and then calculated the Pearson correlation with direction specific performance for each orientation. We repeated this process 10 000 times, thereby obtaining a distribution of r values for α_{1-3} (**FigS4**). We found that in Experiment 3, the correlations for vertical and horizontal orientations indeed represent a true specific relationship, as the obtained true r values were higher than those obtained by correlating these values by chance (horizontal p=.025, vertical p=.037). This was not the case for Exp 1, due to the fact that several participants reached very high performance in all orientations, making the separation of orientation-specific effects difficult, or impossible, if the performance is 100% for all three orientations.

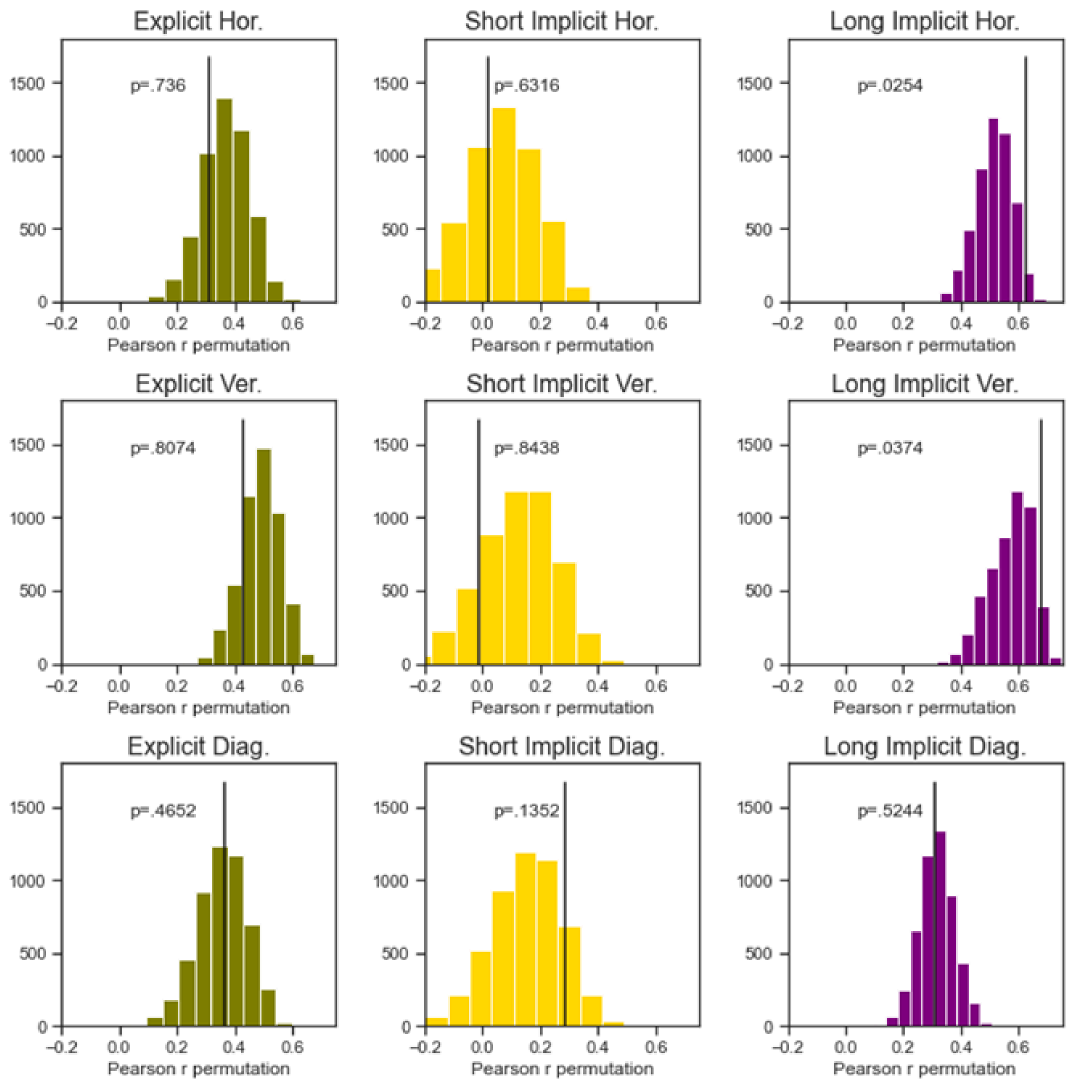

FigS4: **The results of the correlation-specificity analysis.** Each panel shows the obtained true value of correlation (black vertical line), the distribution of shuffled Pearson r values obtained by simulation, and the corresponding

p-value of the obtained value within the shuffled distribution. The nine panels show the results of the analysis arranged for the three orientations of the base-pairs (rows, Top: Horizontal; Middle.: Vertical, Bottom: Diagonal) in each of the three experiments (columns). True specific correlations were found for the horizontal and vertical orientations in the Long Implicit experiment ($p<.05$).

S5: Modeling direction specific statistical influences

For modeling direction specific pair influences on eye-movements, we used a 3-parameter model (with $\alpha_1, \alpha_2, \alpha_3$ as parameters) to predict transition probabilities between cells in the three directions relative to the average gaze transitions behavior (transition probability matrix within cells) in those directions for each participant independently.

For a demonstration of this model, consider the following cell numbering, representing the 3*3-stimulus presentation grid of our experiment:

**Cell 1**	**Cell 2**	**Cell 3**
**Cell 4**	**Cell 5**	**Cell 6**
**Cell 7**	**Cell 8**	**Cell 9**

Using the labels of this table, $p(Cell2|Cell1)_{emp.}$ of a participant defines the empirical transitional probability of moving the gaze from Cell1 to Cell2 over the course of the experiment. Depending on the content of Cell1 on any given trial, we updated these transition probabilities by using $\alpha_1, \alpha_2, \alpha_3$, the three direction-specific parameters of the model the following way:

If on a given trial, shapes in Cell 1 and Cell2 were part of the same *horizontal* pair:

$$p(Cell2|Cell1) = p(Cell2|Cell1)_{emp.} + \alpha_1$$

If on a given trial shapes in Cell1 and Cell4 were part of the same *vertical* pair:

$$p(Cell4|Cell1) = p(Cell4|Cell1)_{emp.} + \alpha_2$$

If on a given trial shapes in Cell1 and Cell5 were part of the same *diagonal* pair:

$$p(Cell5|Cell1) = p(Cell5|Cell1)_{emp.} + \alpha_3$$

Transitions probabilities from Cell1 to other cells remained as before:

p(Cell3|Cell1)= p(Cell3|Cell1)*emp.*

Following the example of Cell 1 above, transition probabilities from all cells that contained shapes were updated with $\alpha_{1\text{-}3}$ on each trial and then renormalized. Transition probabilities from empty cells remained unchanged. The values of $\alpha_{1\text{-}3}$ were fitted on the range 0-1 trial-by-trial by using the *minimize* function of *scipy,* by minimizing the negative log-likelihood over the observed gaze transition sequence of each trial. In this analysis, we only used trials with at least three transition events (≈>90% of trials). Finally, independently for each participant, values of $\alpha_1, \alpha_2, \alpha_3$ were averaged separately across trials, and used as predictors of direction specific pair influences as described in the General Methods of the main text and presented in Fig 4.

S6: Difference between exploratory and confirmatory gaze in the prediction of familiarity test performance.

The trial-by-trial pearson r values for exploratory and confirmatory pair looks and test performance were compared with paired t-test in 36 trial long bins (as on Fig 3). Bins that provide evidence for a difference (based on the bayes factor) are highlighted in red, bins that support the null are highlighted in green, bins with no support for either hypothesis are not highlighted. The presented p-values have not been corrected for multiple comparisons.

	**Exp 1**	**Exp 2**	**Exp 3**
Trials 1 - 36	t_{35}=4.43, *p*<.001, BF=275.118	t_{35}=1.03, *p*=.31 BF=0.293	t_{35}=0.12, *p*=.903, BF=0.18
Trials 37 - 72	t_{35}=4.21, *p*<.001, BF=153.079	t_{35}=0.67, p=.508 BF=0.221	t_{35}= 0.04, *p*=.965, BF=0.179
Trials 73 - 108	t_{35}=2.03, *p*=.05, BF=1.117	t_{35}=0.63, *p*=.532 BF=0.216	t_{35}=1.68, *p*=.102 , BF=0.638
Trials 109 - 144	t_{35}=0.19, *p*=.854, BF=0.182	t_{35}=0.57, *p*=.57, BF=0.209	t_{35}=0.91, *p*=.367 BF=0.264
Trials 145 - 180			t_{35}=1.06, *p*=.295, BF=0.302
Trials 181 - 216			t_{35}=-0.27, *p*= .789, BF= 0.185
Trials 217- 252			t_{35}=0.25, *p*=.801, BF=0.184
Trials 253 - 288			t_{35}=2.21, *p*=.034, BF=1.528

S7: Gaze patterns distributions reflects position and content

We found that the entropy of the gaze transition probability distribution is influenced by both the location on the 3*3 grid) and the content of the cells. To investigate the latter, we separated gaze transitions based on whether a location contained a shape or not on a given trial. We found that gaze transition entropy was higher (all ps<.001), if there was a shape present in a given location in all of the three experiments. This demonstrates that the scanpath is influenced by the presence of shapes.

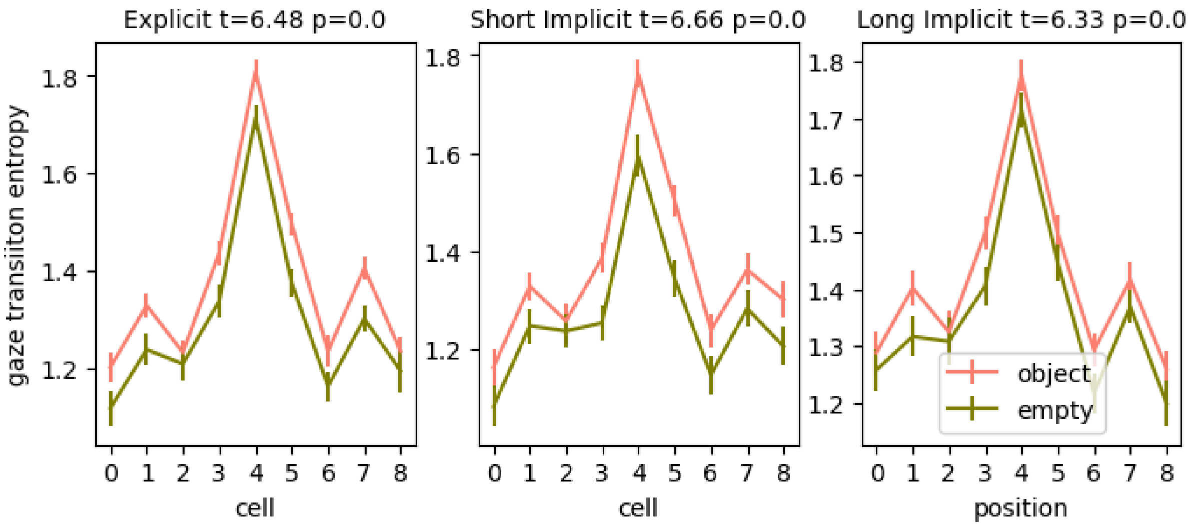

Fig S7A: Gaze transition entropy as a function of location (x-axis, cells are counted from top left=0, top middle=1 .. bottom right=8) and content (containing shape vs empty) in the three experiments. Paired t-test results comparing empty vs shape containing cells (averaged across cells for each participant) are in the titles above.

To investigate the location dependence of the above effect, we calculated the difference in entropy between object containing and empty cells for each cell separately. Next, we averaged this difference across mid vs central locations (**Fig S7B**). We found that this effect was more pronounced at the five central locations opposed to the four corners, where people tended to scan more stereotypically.

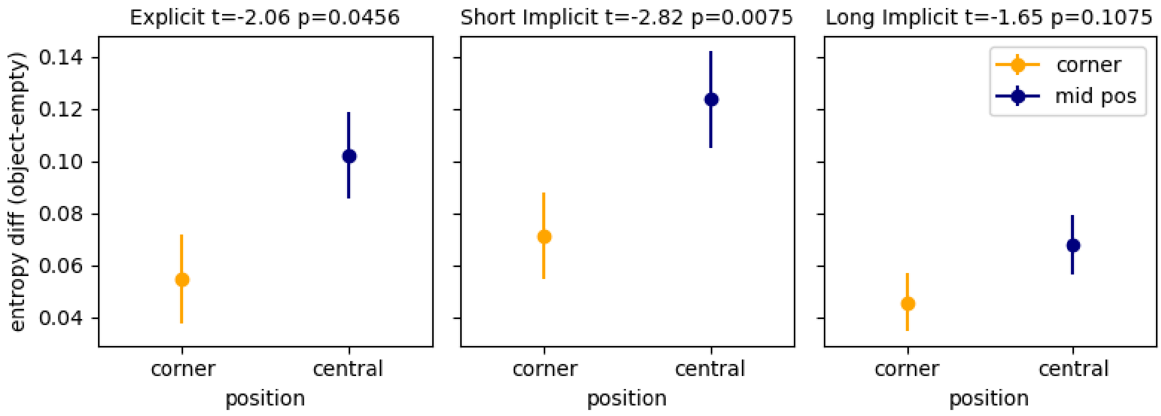

Fig S7B: Position dependence of active learning: the influence of the presented stimuli on the gaze transition entropy was more pronounced at the five central locations, relative to the corners. Results of paired t-tests are in the title for each experiment..

S8: Fixation rates for the best learners

Below, we show changes in within pair fixation rates for participants who performed above 85% on the familiarity test. The data below is averaged across participants for each trial. The effect of learning on eye movements is not necessarily small (as suggested by Fig 2, including data from all participants), as those participants who learn well, show changes of approximately 10-15% in pair gaze rates.

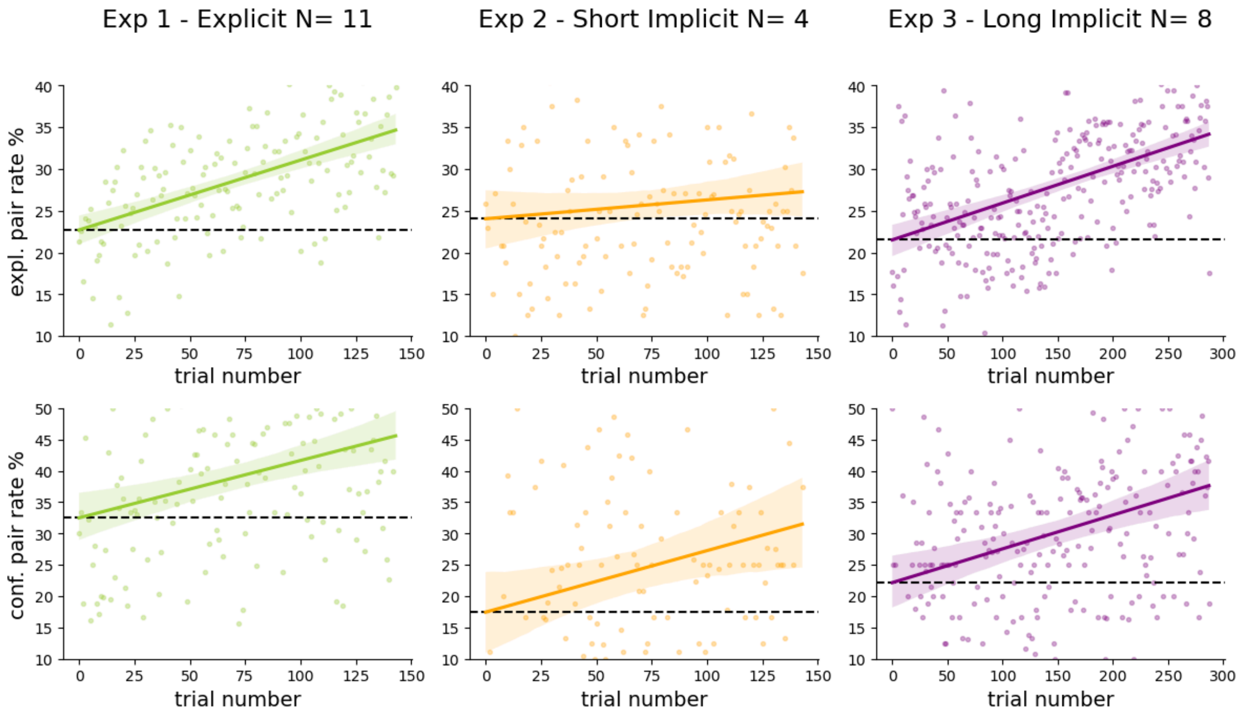

Fig S8: Changes in pair gaze rate for high learners (test performance 85%+) for exploratory (top) and confirmatory (bottom) transitions in the three experiments, with best fit linear regression lines and confidence intervals. Each dot represents a trial averaged across observers.

S9: Gaze contingent methodology

The sampling rate of eye-tracker and the frequency of the display always limit the tightness of the gaze-contingent stimulus presentation, and some delay is inevitable. For example, in Droll et al 2005, the authors measured participants' change detection performance and in such an experiment it is important to update a scene during a saccade to ensure that the visual transient is not detected. However, the objective in our experiment was not to achieve the highest responsiveness of the system with the tightest temporal delay in stimulus presentation by revealing stimuli in response to a single gaze sample as fast as possible. For the success of our present experimental design, the main goal was to encourage participants to reveal their visual sampling strategy. This required designing a procedure with two features. First, participants had to observe the content of the cells carefully and sample new cells by being implicitly aware that sampling has a time cost. We wanted to avoid a design where a quick scan across all the presentation grids would reveal the content of the covered cells so that the observer could collect information about all stimuli in the presented display. Indeed, during testing the early version of the pilot code, we encountered a number of observers following this “scanning” strategy prompting us to make adjustments to the procedure in the opposite direction of higher responsiveness as explained below. Second, we needed to set up the stimulus display so that the observer’s decision as to which piece of information they are interested in next is clearly indicated by their eye movement.

To address the first issue, we only revealed the stimuli if two subsequent gaze samples at 60Hz (16ms apart) were within the gaze contingent region of the same cell of the grid, in which case the screen was updated with the shape information within a time of 50 ms, resulting in a total delay of 50-65 ms from the first gaze sample to stimulus display. This “slowing down” in timing goes against the idea of tightening the gaze-contingent stimulus presentation as much as possible, but it was sufficient to maintain a naturally comfortable feel of wandering to a next location

during exploration and “immediately” seeing the content of the given grid cell while also preventing the use of non-specific exploration strategies by the participants. This claim is in accordance with one of our previous studies, in which we successfully used a delay of 120 ms to investigate gaze contingent visual exploration (Hoppe & Rothkopf, 2019).

However, we still had to make sure that the “slowing down” would not introduce another problem: quick saccades away from a cell while the stimulus in the cell is being revealed. To this end, we measured the gaze transition events in Exps 1 & 2, out of which we recorded on average 1223.49 +/- 259.15 transitions during the 144 trials across participants. Only about 31.1 +/- 16.77 of these happened within 50 ms after triggering a display of a stimulus following the second fixation in the same cell, which amounts to 2.5% of gaze transition events. This small fraction confirms that the observers overwhelmingly followed the pattern of “fixating – seeing the shape – moving on” during their explorations. In other words, the delay in our designs, indeed, found a sweet-spot compromise between the complementary requirements of the task.

Droll, J. A., Hayhoe, M. M., Triesch, J., & Sullivan, B. T. (2005). Task demands control acquisition and storage of visual information. *Journal of Experimental Psychology: Human Perception and Performance*, *31*(6), 1416.

Hoppe, D., & Rothkopf, C. A. (2019). Multi-step planning of eye movements in visual search. *Scientific reports*, *9*(1), 144.
